# What programmatic assessment in medical education can learn from healthcare

**DOI:** 10.1007/s40037-017-0345-1

**Published:** 2017-04-10

**Authors:** L. Schuwirth, C. van der Vleuten, S. J. Durning

**Affiliations:** 1 Prideaux Centre for Research in Health Professions Education, School of Medicine, Flinders University, Adelaide, South Australia Australia; 2 Department of Educational Development and Research, Maastricht University, Maastricht, The Netherlands; 3 Department of Medicine and Pathology, F. Edward Hébert School of Medicine, Uniformed Services University, Bethesda, USA

## Background

A new approach to assessment is emerging in medical education, called programmatic assessment. Programmatic assessment is an approach in which routine information about the learner’s competence and progress is continually collected, analyzed and, where needed, complemented with purposively collected additional assessment information, with the intent to both maximally inform the learner and their mentor and allow for high-stakes decisions at the end of a training phase. For this, a variety of assessment instruments are usually used [1–3]. Programmatic assessment is being used in various medical school settings around the world and it is also becoming more popular in graduate medical education and continuing professional development [4–6]. Programmatic assessment is quite different from more traditional assessment programs with the typical ‘module-test’ building blocks focussing almost entirely on assessment *of *learning. We think that programmatic assessment actually makes more sense from various perspectives and we want to use analogies with clinical medicine to explain why we think so. Therefore, we will begin with a brief description of the programmatic assessment approach and then we will use analogies to help explain the rationale behind it. We do not intend to use these analogies as evidence for the validity of programmatic assessment as an assessment approach – there is an emerging body of research with that remit – but we merely seek to explain the concepts of programmatic assessment using a more medical narrative.

In the programmatic assessment approach, each assessment produces meaningful feedback to the learner. This feedback may be quantitative, qualitative or both. Each individual assessment is not initially meant for ‘high-stakes’ decision-making, but has to be used by the learner to analyze their own performance, formulate concrete learning goals and demonstrably attain them. Individual assessments are used as components that are to be collected, for example in a portfolio, and then analyzed by a faculty member or committee into a rich diagnostic picture that will allow defensible high-stakes decisions. Typically, all information is periodically reviewed by an assessment committee for summative decisions, combining information from various sources in a way that is meaningful by content [1, 2, 7]. So, for example, results on parts of a multiple-choice examination may be combined with parts of a mini-CEX or OSCE examination to draw conclusions as to the examinee’s progress in a domain of performance. Based on this review, remediation plans are provided. A continuous dialogue between the learner and a dedicated staff member (called either mentor, supervisor or coach in different institutes) further scaffolds the focus on feedback, analysis of competence development, remediation and personal development. Instead of a conventional assessment such as taking a high-stakes multiple-choice examination followed by a pass-fail decision, programmatic assessment addresses both the attained competence levels and their developmental processes. There are similarities with normal practice in healthcare and therefore, in this paper, we will present five analogies between programmatic assessment and clinical healthcare to explain the concepts behind this approach.

## Analogies

### Analogy 1: Like the concept ‘health’, the concept of competence may be difficult to define but it can be evaluated, promoted and improved

The WHO definition of health is: ‘a state of complete physical, mental, and social well-being and not merely the absence of disease or infirmity’ [8]. A popular definition of competence is ‘the habitual and judicious use of communication, knowledge, technical skills, clinical reasoning, emotions, values, and reflection in daily practice for the benefit of the individual and the community being served’ [9]. Both definitions convey an understanding, but do not really help in actual practice. The definition of health does not help in diagnosing and treating specific patients and the definition of competence is not helpful for the assessment and education of specific learners at any level (students, residents, or physicians in practice). What they have in common, though, is that despite the fact that both ‘health’ and ‘competence’ are almost impossible to define, they can still be evaluated and improved. Doctors are able to diagnose ‘ill-health’, and likewise it is possible for expert assessors to diagnose ‘dyscompetence’ [10]. Of course there are also differences in that in most domains of healthcare (but less so in mental health) the illness may have directly observable clinical features whereas competence always has to be inferred from what we can observe. But, on the other hand, the analogy can be extended in that much like there is no single instrument that will diagnose any illness in its full scope, there is no single instrument that will assess ‘competence’ in its entirety. Diagnosing in healthcare involves the careful collection and collation of information from various sources such as the history, physical examination, lab tests, pathology or radiographic studies. Likewise, programmatic assessment is the careful collection and collation of information from various sources not only to diagnose the examinee’s competence but also to promote and improve their competence.

### Analogy 2: Merely using structured and standardized testing in assessment is like diagnosing a patient on lab-values alone

A commonly debated topic in traditional assessment models relates to which information best to use: quantitative or qualitative; with quantitative approaches being given priority for higher stakes assessments due to their psychometric properties. The debate in programmatic assessment, however, is not whether quantitative information is better than qualitative or vice versa, but how best to combine them for each individual learner. A healthcare system that is based purely on lab testing would not be optimal but neither would a system that has no access to lab testing. In assessment, the inclusion of qualitative information often raises concerns that unstructured assessments are not of the same rigour as the standardized tests, because the latter can be quality assured with psychometrics and the former cannot [11]. But, again, the analogy with healthcare practice is powerful. When we order a haemoglobin level for a patient we are generally not interested in the lab analyst’s opinion about the haemoglobin level but merely want to know the numerical value, for example 12.2 g/dl (7.57 mmol/l). The reliability (and validity) of the measurement are determined by characteristics such as the quality of the lab equipment, population data and 95% confidence intervals. When, on the other hand, we order a histopathology report we are not interested in receiving a number but we want the pathologist’s expert opinion. The reliability (and validity) of that outcome is determined by the cogency of the report with respect to the clinical questions, the trust in the education of the pathologist, the plausibility of his/her conclusions and their careful documentation. It is nonsensical to apply the measurement-type quality criteria to this report or the credibility criteria to the lab value. In a programmatic assessment program quality assurance of the assessments likewise cannot be purely based on psychometrics and will have to incorporate careful documentation, cogent underpinnings of decisions and assessment expertise development [12].

At the national level the analogy also holds. Programmatic assessment is by no means an argument against national testing. National healthcare systems often benefit from national screening programs provided there is suitable diagnostic follow-up. So would a programmatic assessment benefit from including the results on national testing, provided there is an equivalent suitable ‘diagnostic’ follow-up of those examinees who underperform at this level, and questions like: ‘What is the prevalence of the disease ’dyscompetence‘?’, ‘Is the screening tool sensitive/specific enough?’, ‘Is the outcome of the disease with screening better than without?’ and ‘What is the number needed to treat/harm?’ are addressed.

### Analogy 3: Testing alone is not enough to effectively lead to higher competence levels of learners like merely making a diagnosis is not enough to cure a patient

The typical aphorism to express this concept is the statement that ‘merely taking a patient’s temperature is not enough to cure them’. Purely diagnosing a patient, in itself, is not enough; it has to be combined with therapeutic actions.

Assessment also needs to be ‘therapeutic’. If diagnostic procedures in healthcare were to just result in a ‘healthy/not healthy’ decision, the clinician would have very little to act on to determine his/her therapeutic plan. This is why in healthcare, diagnostic work-ups are typically purposefully planned to diagnose exactly what is wrong, how severe the condition is, and what the best course of action is. It is unlikely that a clinician would simply run all the tests again a couple weeks later to see whether they have normalized without any therapy. Yet in traditional testing this is often what happens with re-examinations which are often merely a repeated measurement of the original test. Further, these tests are not optimally informative as to potential underlying causes of the problem – they offer limited strategies for remediation in and of themselves. Of course, one cannot ignore the effects of tests on learning – in particular the test-enhanced learning effect [13, 14] – but the reliance on these effects without targeted information is likely to make the whole process less effective as the importance of the role of feedback and targeted practice – deliberate practice – is generally accepted [15].

In programmatic assessment, the combination of different types of information is deliberately used to inform the learner and faculty alike about what specific remedial activity would be needed. For example, the results on multiple choice questions (from a larger test) on abdominal anatomy can be combined with those on an OSCE station on abdominal examination and a mini-CEX with a patient with abdominal pain to determine whether the learner has insufficient technique (and therefore just requires more practice), insufficient anatomical knowledge (and therefore requires remediation in that domain) or insufficient patient consultation skills. This makes much more sense than compensating poor performance on an OSCE station on, for instance, abdominal examination with good performance on an OSCE station on ‘knee examination’. Again, this way of combining information is the norm in healthcare; a clinician would never tell a patient that unfortunately their Hb level is too low but fortunately their glucose is too high and so, on average, their lab values are ok. The clinician would combine the glucose level with complaints about fatigue, polydipsia and polyuria and absent arterial pulses with poor wound healing to make sense of the information (both diagnosis and treatment of a specific problem) rather than to merely mathematically average it.

### Analogy 4: Like diagnosing a disease is not merely a tick box exercise ‘diagnosing’ dyscompetence using a tick box exercise does not work either

Currently, various educational and licencing organizations have published outcomes in terms of ‘roles’ or ‘competencies’. Invariably they have divided these competencies further into more detailed sub-competencies or at even deeper levels of detail (‘sub-sub-competencies’). From an assessment point of view, this is often seen as problematic because of two reasons.

First, there is a general feeling that it is never enough. Medicine seems to be an almost infinite domain and there are always other items that can be added to the list of sub-competencies, often leading to extensive discussions about what to include and what to leave out. This is not only true for licencing bodies but also for medical schools in determining the content of their curriculum.

Second, the ‘whole’ has to be more than the sum of the ‘parts’. So, when dyscompetence is dissected into lists of separate detailed items, finding a proper way to recombine them – to glue them back together again – in order to assess ‘dyscompetence’ is a real challenge. It is clear that a checklist approach, expecting the competent candidate to tick all the items on the extensive lists will not work in most cases.

Here too, an analogy with healthcare can be helpful. In healthcare, every diagnosis can be described in signs and symptoms, and textbooks often provide long lists of signs and symptoms for each diagnosis. But, a patient does not have to have them all to be diagnosed with a certain illness; there is no need to ‘tick all the items’. In most cases the expert clinician makes an integral ‘gestalt’ diagnosis, and is able to verbalize the signs and symptoms and his/her evaluation to explain their rationale, as a top-down processing activity [16]. Without this ability for gestalt diagnosis, the clinician would have to know all the exact positive and negative predictive values of all signs and symptoms for each diagnosis in the differential diagnosis, and do the complex mathematics mentally to produce the most likely diagnosis as a complete bottom-up processing. Yet, the clinician has had the opportunity to develop this expertise through years of training, with the use of heuristics and guidelines, with guided experience and a gradual descaffolding. The same would have to apply to assessors. Ideally a similar approach to the development of assessment literacy would be applied to assessors [17].

But even with the gestalt-type, top-down processing, individual signs and symptoms are very useful; they are needed to describe, evaluate and actually help improve the patient’s health status. In line with our first analogy between ‘illness’ and ‘dyscompetence’, the long lists of competencies, sub-competencies and even sub-sub-competencies are not trivial, but they are to be used as the equivalent of signs, symptoms and findings to describe, evaluate and improve the learner’s dyscompetence. Of course, a learner can be highly competent despite not all sub-competencies being met or even assessed, much like a patient can have a certain diagnosis without all the symptoms and findings being present or being diagnosed. So instead of using competency frameworks as checklists they are probably better used to explain and support the assessors’ expert gestalt judgements.

### Analogy 5: Healthcare and assessment systems both rely on expert practitioners that must be developed and nurtured

It is clear that no medical organization can function without the expertise of its healthcare staff. Although standardization and structuring in procedures in the organization has been very beneficial to the quality of healthcare – evidence-based medicine for example has been instrumental in ensuring that medical decisions are more evidence informed – they do not provide a substitute for expertise. A good format for a patient chart is helpful when it supports the clinician in doing a consultation but the form in itself does not replace the physician’s expertise. Moreover, as with the Hb example, data collection can be done objectively, but data interpretation never is. The same Hb level can be cause for concern in one patient and reason for optimism in another.

The same applies to assessment; a common myth with the traditional approach is that assessment should be objective, but assessment actually never is. Even the most structured multiple choice examination is the result of numerous human judgements: what topics to cover, the division of topics among the test – the blueprinting –, the actual items to include, the wording of the items, etc. It is only the final part, the data collection, which is objectified. Young children would be able to hand out the forms, take in the answer sheets and even calculate the scores, simply because all the subjective expert judgments have been used in the production of the test paper. With workplace-based assessment it is exactly the other way around, the expertise is needed when the observation is made. The specific design of the form is relatively unimportant as long as it facilitates the expert examiner in their task. Clearly we would not dream of having our young children perform a workplace-based assessment, or even an adult with no specific expertise.

The bottom line is that in every type of assessment expert human judgement – from various health professional domains – is needed and this judgement is only valid and reliable if it is based on sufficient content and assessment expertise [12, 18]. It must be supported by an organization that will effectively and efficiently support and facilitate the expert and that has procedures in place to ensure carefulness, transparency, documentation, and inter-collegial consultations. Therefore, such an organization will need to devote resources to staff development and development of assessment expertise. The ultimate corollary of this is that assessment is not merely a measurement problem, but an educational design and staff development issue.

## Conclusion

With these analogies we have tried to illustrate the thinking behind current developments in assessment and how it is actually highly informed by the thinking in healthcare. One of the analogies we have not discussed in length is the one with continuity of care. For optimal development of competence, as with health, longitudinality is important and hand-overs for example between echelons are essential. Hand-over without an informative document is nearly impossible and so would be the ‘hand-over’ between stages of training. From undergraduate to postgraduate to continuing medical education, programmatic assessment would be the conduit through which competence development is monitored and optimized. But, to be frank, here is where programmatic assessment as an educational concept has to come to grips with the practical context. Despite the successful implementations of programmatic assessment around the world, this is the aspect that should be put high on the agenda [5, 7]. The reason for this lies precisely in the aspects in which the analogy between programmatic assessment and healthcare fails. The most important difference is the different cultures; in healthcare, patients generally see their doctor as their supporter but in assessment learners often see their examiner as their opponent. Also, patients are used to the practice of healthcare as a diagnostic and therapeutic process and it is what they expect when they consult a doctor. The experiences with education of trainees, learners and teachers on the other hand, have been quite different from programmatic assessment and therefore their ideas about what constitutes normal assessment will need to change before PAL can be implemented. This is why we hope that these analogies between programmatic assessment and healthcare, limited as they may be, can help in developing a more common language between assessment developers and supervisors/students/trainees. We hope further that such a shared language would stimulate their involvement in the assessment process, much like involving patients in management through shared decision making.
